# Development of a simple prediction model for adrenal crisis diagnosis

**DOI:** 10.1038/s41598-020-70466-4

**Published:** 2020-08-11

**Authors:** Takuyuki Katabami, Hidekazu Tsukiyama, Makito Tanabe, Ren Matsuba, Mariko Murakami, Ami Nishine, Sachi Shimizu, Kensuke Sakai, Yasushi Tanaka, Toshihiko Yanase

**Affiliations:** 1 Division of Metabolism and Endocrinology, Department of Internal Medicine, St. Marianna University School of Medicine Yokohama City Seibu Hospital, 1197-1, Yasashicho, Asahi-ku, Yokohama, Kanagawa 241-0811 Japan; 2 Division of Metabolism and Endocrinology, Department of Internal Medicine, St. Marianna University School of Medicine, Kanagawa, 216-8511 Japan; 3 Department of Endocrinology and Diabetes Mellitus, Fukuoka University Hospital, Fukuoka, 814-0180 Japan; 4 Center of Life-Style Disease, St. Marianna University School of Medicine Toyoko Hospital, Kanagawa, 211-0063 Japan; 5 Division of Metabolism and Endocrinology, Department of Internal Medicine, St. Marianna University School of Medicine Kawasaki Municipal Tama Hospital, Kanagawa, 214-8525 Japan; 6 Muta Hospital, Fukuoka, 814-0163 Japan; 7 Fukuoka University, Fukuoka, 814-0180 Japan

**Keywords:** Biomarkers, Signs and symptoms, Endocrinology, Endocrine system and metabolic diseases

## Abstract

To develop a prediction model for adrenal crisis (AC) diagnosis among individuals with adrenal insufficiency that relies on the values of routinely measured clinical parameters, for application in standard clinical practice. We retrospectively analysed data from five referral centres in Japan. Multivariate binary logistic regression was used to identify independent predictors of AC, and receiver operating characteristic curve analysis was used to determine their optimal cut-off points. The analysis included data from 54 patients with 90 AC events. Logistic regression revealed that serum sodium and C-reactive protein (CRP) levels were independent predictors of AC. Serum sodium levels < 137 mEq/L had a sensitivity of 71.1% and specificity of 95.6%. CRP levels > 1.3 mg/dL had a sensitivity of 84.4% and specificity of 94.9%. In combination, serum sodium levels < 137 mEq/L or CRP levels > 1.3 mg/dL for AC diagnosis had sensitivity and specificity values of 97.8% and 94.4%, respectively. The combined use of serum sodium and CRP levels had high sensitivity and specificity, and can be used for AC screening in standard clinical practice. The model can assist in identifying AC among high-risk individuals. A larger prospective study is needed to validate these results.

## Introduction

In individuals with chronic adrenal insufficiency (AI), adrenal crisis (AC) is a serious and life-threatening event, even among those with steroid replacement therapy^1^. The mortality of individuals with chronic AI is higher than that of the general population, with the associated mortality risk shown to be approximately 2.5-fold higher^1,2^. Epidemiological studies have shown that the AC incidence among individuals with AI who are receiving standard replacement therapy is 6–8% per year^3,4^. Among the important risk factors for AC is a previous episode of AC^5^. A prospective study showed that the AC-related mortality value was 0.5 deaths/100 patient-years^6^. As no consensual definition of AC exists, a physician must diagnose AC based on the presence of non-specific symptoms, signs, and/or the results of routine laboratory tests. Furthermore, some endocrine tests such as rapid assays for cortisol and adrenocorticotropin (ACTH) are available only in a limited number of medical institutes. AC diagnosis is challenging and there is a need for a rapid, widely available sensitive method for the same. Therefore, the purpose of this study was to develop a prediction model for AC diagnosis that relies on the values of standard biochemical tests and can be applied in standard clinical practice.

## Results

### Baseline patient characteristics and precipitating factors for AC

In total, 90 events in 54 patients with AC were included in the analysis (Table 1). Twenty-one (38.9%) patients experienced more than one AC event during the data collection period (twice, N = 13; three times, N = 4; more than three times, N = 4). The mean age at admission for AC was 62.8 ± 1.7 years (range, 29–91 years) and approximately half of the patients were women. Twenty-four patients were diagnosed with AI for the first time after AC onset and the remaining 30 were previously diagnosed with AI; 66 (73.3%) of the 90 events were observed in the latter group of patients. Of 54 patients with AC, 16 (29.6%) had primary AI and 38 (70.4%) secondary AI. The underlying aetiology of AI is summarised in Table 2. The causes of primary AI were hypoadrenalism owing to steroid synthase inhibitor (metyrapone, N = 3; mitotane, N = 2; trilostane, N = 1) use, bilateral adrenalectomy (N = 5), and Addison’s disease (N = 5). The most commonly observed causes of secondary AI were isolated ACTH deficiency (N = 9), postsurgical hypopituitarism (N = 8), and steroid withdrawal syndrome (N = 7). Furthermore, one patient developed AC during treatment with pembrolizumab, which is a humanised immunoglobulin G4-κ monoclonal antibody against programmed cell death 1 with potential immune checkpoint inhibitory and antineoplastic activities.

**Table 1:** Characteristics of the patients with adrenal crisis at baseline.

Total number of patients (events)	54 (90)
Sex, male:female, frequency	26:28
Age (years) at onset of the AC event, mean ± standard deviation	62.8 ± 1.7
Multiple hospitalisations, number of patients (events)	21 (59)
**AI status at the time of the AC, number of patients (events)**	
Undiagnosed	24 (24)
Diagnosed	30 (66)
**AI subtype, number of patients (events)**	
Primary AI	16 (26)
Secondary AI	38 (64)

AC, adrenal crisis; AI, adrenal insufficiency.

**Table 2:** Etiology of adrenal insufficiency among the study patients.

Etiology	Number of patients
**Primary AI**	
Steroid synthase inhibitors^a^	6
Bilateral adrenalectomy	5
Addison’s disease	5
**Secondary AI**	
Isolated adrenocorticotropic hormone deficiency	9
Postsurgical hypopituitarism	8
Steroid withdrawal syndrome	7
Sheehan’s syndrome	4
Idiopathic panhypopituitarism	4
Hypothalamic hypopituitarism	2
Post cushing’s syndrome surgery	2
Autoimmune hypophysitis	1
Pembrolizumab	1

^a^Steroid synthase inhibitors: metyrapone (N = 3), mitotane (N = 2), and trilostane (N = 1).

AI, adrenal insufficiency.

The precipitating factors for AC are shown in Table 3. The most frequently noted precipitating factor for AC was the presence of infectious diseases including gastroenteritis (primary, 19 events; secondary, 44 events) in both AI subtypes. The cessation or inadequate dose reduction of glucocorticoid replacement by patients (five events) or attending physicians (three events) was another precipitating factor in patients with secondary AI. In some cases, no underlying factor could be identified (primary AI, two events; secondary AI, 11 events). Other causes included gastrointestinal bleeding, duodenal ulcer, advanced cholangiocarcinoma, use of anticonvulsants, vomiting during chemotherapy, and post-traumatic stress (one event each).

**Table 3:** Precipitating factors for adrenal crisis.

Precipitating factor	Primary AI (n = 26)	Secondary AI (n = 64)
	No. of events (%)	No. of events (%)
Infectious disease, including gastroenteritis	19 (73.1)	44 (68.8)
Cessation or inadequate dose reduction of glucocorticoid replacement	0	8 (12.5)
By the patient	0	5 (7.8)
By the attending physician	0	3 (4.7)
Others^a^	5 (19.2)	1 (1.6)
Unknown	2 (7.7)	11 (17.2)

^a^Other causes included gastrointestinal bleeding, duodenal ulcer, advanced cholangiocarcinoma, use of anticonvulsants, vomiting during chemotherapy and post-traumatic stress (one event each).

AI, adrenal insufficiency.

### Changes in blood pressure and laboratory findings after AC treatment

The interval between AC onset and blood sampling at the chronic phase was 175.9 ± 9.4 (range: 13–359) days. Serum sodium levels, estimated glomerular filtration rate (eGFR), and systolic blood pressure (BP) were significantly lower (*P* < 0.001, *P* = 0.01, and *P* < 0.001, respectively), and serum potassium level, serum creatine level, and C-reactive protein (CRP) level were significantly higher (*P* = 0.005, *P* < 0.001, and *P* < 0.001, respectively) in the acute phase than in the chronic phase (Table 4, left column). Plasma glucose and haemoglobin (Hb) levels in each phase were similar. However, in patients with hypoglycaemia and/or anaemia in the acute phase, plasma glucose and Hb levels in the chronic phase were markedly higher than those in the acute phase (plasma glucose, 53.8 ± 3.4 mg/dL to 99.0 ± 10.3 mg/dL, *P* < 0.001; Hb, 10.6 ± 0.23 mg/dL to 11.7 ± 0.22 mg/dL, *P* < 0.001) (Table 4, right column).

**Table 4:** Changes in the parameters between the acute (left column) and chronic phases (right column).

	All events (N = 90)			Events showing abnormal value at acute phase based on reference range			
	Acute phase (Mean ± SD)	Chronic phase (Mean ± SD)	*P* value	No. of events	Acute phase (Mean ± SD)	Chronic phase (Mean ± SD)	*P* value
Serum sodium level (mEq/L)	132.1 ± 0.97	140.5 ± 0.20	< 0.001	43	125.2 ± 1.40	140.1 ± 0.31	< 0.001
**Serum potassium level (mEq/L)**							
All cases of AC	4.3 ± 0.07	4.1 ± 0.04	0.005	10	5.6 ± 0.20	4.3 ± 0.11	< 0.001
AC due to primary AI	4.4 ± 0.17	4.2 ± 0.06	0.247	4	6.0 ± 0.49	4.6 ± 0.0	0.070
AC due to secondary AI	4.3 ± 0.07	4.1 ± 0.06	0.005	6	5.4 ± 0.07	4.2 ± 0.15	< 0.001
Plasma glucose level (mg/dL)	106.3 ± 6.95	104.9 ± 3.29	0.147	19	53.8 ± 3.40	99.0 ± 10.3	< 0.001
**Hemoglobin level (g/dL)**							
Male	12.6 ± 0.37	12.3 ± 0.26	0.483	24	11.2 ± 0.31	11.8 ± 0.28	0.092
Female	11.9 ± 0.30	12.4 ± 0.22	0.124	18	9.8 ± 0.27	11.5 ± 0.34	0.003
Serum creatinine level (mg/dL)	1.2 ± 0.09	1.0 ± 0.12	< 0.001	53	1.52 ± 0.21	1.04 ± 0.06	0.008
eGFR (mL/min/1.73 m^2^)	57.2 ± 4.24	62.3 ± 3.14	0.010	54	35.30 ± 1.87	49.7 ± 2.36	< 0.001
C-reactive protein level (mg/dL)	10.3 ± 1.00	0.3 ± 0.08	< 0.001	81	11.6 ± 0.92	0.34 ± 0.72	< 0.001
Systolic BP (mmHg)	107.5 ± 3.61	122.2 ± 2.43	< 0.001	30	92.2 ± 3.24	123.6 ± 17.8	< 0.001

Results derived from all events are indicated in the left column and those derived from events showing an abnormal value in the acute phase based on a reference range in the right column. Data are presented as mean ± standard error. *P* < 0.05 based on the Wilcoxon signed rank test or the paired t-test was considered statistically significant. Except for potassium, all parameters were judged as normal based on the reference ranges of each study hospital. Hyponatraemia was defined as a serum sodium level < 136 mEq/L; hypoglycaemia was defined as a plasma glucose level < 70 mg/dL; sex-specific reference values were used for the determination of anaemia (men: < 13.7 g/dL, women: < 11.2 g/dL). The serum potassium level for hyperkalaemia was set at > 5.0 mEq/L, which has been reported to be useful in the diagnosis of Addison’s disease. Hypotension was defined as a systolic blood pressure < 100 mmHg.

AC, adrenal crisis; AI, adrenal insufficiency; BP, blood pressure; eGFR, estimated glomerular filtration rate; SD, standard deviation.

### Development of the prediction model

**Figure 1:**
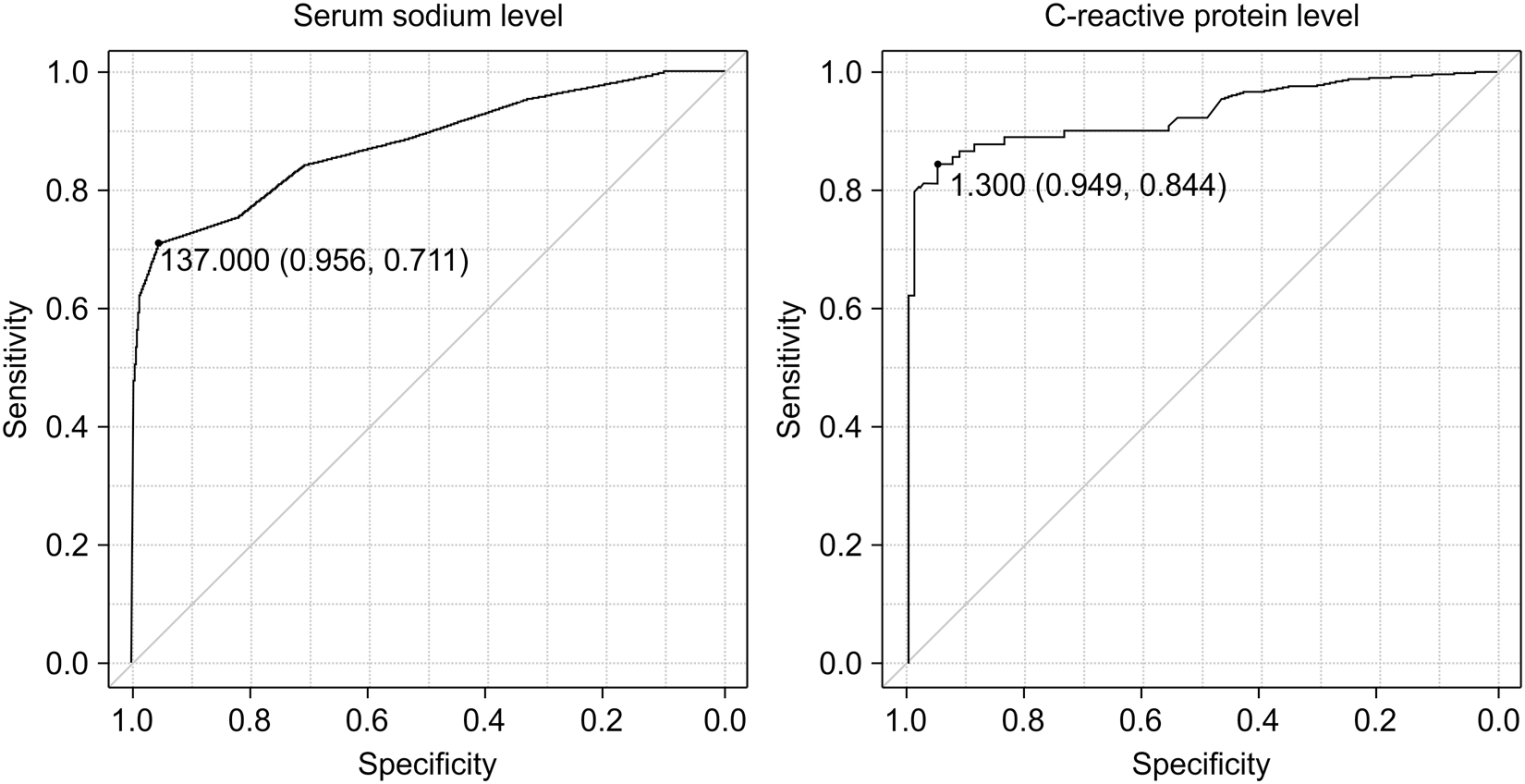
Receiver operating characteristic curves for the determination of the cut-off serum sodium and C-reactive protein level. The optimal cut-off value for serum sodium was 137 mEq/L (sensitivity: 71.1%; specificity: 95.6%; AUC: 0.88 [95% confidence interval: 0.83–0.93]). The optimal cut-point value for serum C-reactive protein was 1.30 mg/dL (sensitivity: 84.4%; specificity: 94.9%; AUC: 0.93, [95% confidence interval: 0.891–0.97]). Abbreviation: AUC, area under the curve.

The five factors (systolic BP and serum sodium, serum potassium, serum creatine, and CRP levels) showing significant differences between the two phases were included in the multivariate logistic regression models. Binary logistic regression analysis was performed to identify independent clinical parameters that were significantly related to the odds of having AC during the acute phase. In the binary multivariate regression analysis, serum sodium (odds ratio [OR], 0.385; 95% confidence interval [CI], 0.20–0.74; *P* = 0.004) and CRP (OR 2.76, 95% CI 1.42–5.34, *P* = 0.003) levels were associated with the presence of AC (Table 5). Furthermore, we performed receiver operating characteristic (ROC) curve analysis to determine the best cut-off serum sodium and CRP levels in AC prediction (Fig. 1). The ROC analysis revealed that a cut-off point of 137 mEq/L for serum sodium levels provided the optimal balance between sensitivity and specificity for use in AC diagnosis (sensitivity: 71.1%, specificity: 95.6%, area under the curve [AUC]: 0.88 [95% CI: 0.83–0.93], Table 6). The analysis also revealed that a CRP cut-off point of 1.3 mg/dL provided the optimal balance between sensitivity and specificity for use in AC diagnosis (sensitivity: 84.4%, specificity: 94.9%, AUC: 0.93 [95% CI, 0.89–0.97]).

**Table 5:** Predictors of acute adrenal crisis determined by binary logistic regression analysis.

	B	β	Odds ratio	95% CI	*P* value
Serum sodium level (mEq/L)	− 0.94	− 8.28	0.39	0.20–0.74	0.004
Serum potassium level (mEq/L)	− 1.73	− 1.05	0.19	0.19–1.64	0.132
C-reactive protein level (mg/dL)	1.03	8.72	2.76	1.42–5.34	0.003
Serum creatinine level (mg/dL)	0.28	0.33	1.19	0.58–2.48	0.632
Systolic blood pressure (mmHg)	− 0.03	− 0.58	0.99	0.93–1.05	0.644

	Odds ratio	95% CI	*P* value		
Serum sodium level (mEq/L)	0.39	0.20–0.74	0.004		
Serum potassium level (mEq/L)	0.19	0.19–1.64	0.132		
C-reactive protein level (mg/dL)	2.76	1.42–5.34	0.003		
Serum creatinine level (mg/dL)	1.19	0.58–2.48	0.632		
Systolic blood pressure (mmHg)	0.99	0.93–1.05	0.644		

The odds ratios were determined using multivariate binary logistic regression. The values of the following variables differed significantly according to the phase: Serum sodium, potassium, C-reactive protein and creatinine, and systolic pressure were chosen as the explanatory variables. The analysis revealed that serum sodium and serum C-reactive protein levels were significant independent risk factors for adrenal crisis diagnosis. *P*-values < 0.05 were considered statistically significant.

B, partial regression coefficients; β, standardized partial regression coefficients; CI, confidence interval.

**Table 6:** Diagnostic parameters of each predictor for the detection of adrenal crisis.

Predictor	Sensitivity (%)	Specificity (%)	PPV (%)	NPV (%)	AUC (95% CI)
(A) Serum sodium level < 137 mEq/L	71.1	95.6	94.2	76.8	0.88 (0.83–0.93)
(B) C-reactive protein level > 1.30 mg/dL	84.4	94.9	94.9	85.9	0.93 (0.89–0.97)
(C) Predictor (A) or (B)	97.8	94.4	94.6	97.7	0.96 (0.93–0.99)

Sensitivity, specificity, positive predictive value (PPV), negative predictive value (NPV) and area under the curve (AUC) (95% confidence interval) are shown. Criteria: (A) Serum sodium level < 137 mEq/L; (B) C-reactive protein level > 1.30 mg/dL; and (C) Serum sodium level < 137 mEq/L and/or C-reactive protein level > 1.30 mg/dL.

To improve the diagnostic accuracy of the prediction model that employed serum sodium and CRP levels, we calculated the sensitivity and specificity of a combination of either a serum sodium level < 137 mEq/L or CRP level > 1.3 mg/dL for AC diagnosis. This criterion had a sensitivity of 97.8% and specificity of 94.4%.

## Discussion

In the present study, we found that the use of serum sodium and CRP in combination had high sensitivity and specificity and may be feasible for AC screening in standard clinical practice.

AC is a life-threatening condition caused by an abrupt deficiency of glucocorticoid, which is required to maintain homeostasis^7^. The annual incidence of AC among individuals with AI is 6–8%^5^. A substantial proportion of educated individuals with AI develop AC too. Among individuals with AC, the associated mortality may be as high as 6%^4,6^. In addition, rapid assays for cortisol and ACTH are only available in a limited number of medical institutes; therefore, simple biochemical methods for AC diagnosis are needed. However, to the best of our knowledge, no clinical studies have focused on the development of simple biochemical diagnostic methods. To address the need for simple diagnostic methods in such settings, we aimed to develop a prediction model for AC. We selected predictors that could be rapidly determined with a high feasibility and that were relevant to AC. For example, although hypercalcaemia and/or eosinophilia appeared to be good candidates^5,6^, they were excluded because their levels are not generally measured in routine clinical practice. Consequently, in this study, we assessed serum sodium, serum potassium, plasma glucose, haemoglobin, serum creatinine and CRP levels, eGFR and systolic BP as possible predictors. We clearly demonstrated that the measurement of serum sodium and CRP levels allowed for the identification of AC presence with a high level of diagnostic accuracy.

Although hyponatraemia is a distinguishing feature of AC, the detection rate using the lower limit of the reference ranges from 9 to 50%^8–10^. In agreement with the results of previous studies, in our study, the prevalence of hyponatraemia based on the reference range of each centre was 47.8%. However, if the optimal cut-off serum sodium level (< 137 mEq/L) calculated using ROC analysis was used, the sensitivity for AC diagnosis exceeded 70% and specificity was adequate. In this context, even when the serum sodium level in patients with a high risk of AC is low-normal or slightly reduced, physicians should suspect AC presence. Renal impairment may cause hyponatraemia; however, we found that in the chronic phase sodium levels tended to be normal and that the decreased sodium level observed in the acute phase may be due to glucocorticoid and/or mineralocorticoid deficiency.

Surprisingly, CRP was the most sensitive biomarker for AC in this study. Although an elevation in CRP levels in patients with AC may be related to an infection-associated change or a non-specific change, an interaction between the hypothalamus-pituitary-adrenal (HPA) axis and proinflammatory cytokines such as interleukin-1, interleukin-6, and tumour necrosis factor-α is a well-known feature of AC^11^. Proinflammatory cytokines activate the HPA axis, and conversely, excessive reactive glucocorticoid secretion has an anti-inflammatory effect that helps in homeostasis maintenance. Thus, a lack of suppressive glucocorticoid activity can enhance the sensitivity of these cytokines^19^, resulting in CRP induction. The optimal CRP cut-off level in the current study was relatively low, suggesting that CRP level increases may not occur solely as a result of infection. In fact, a high CRP level without any obvious infection was observed in 25.6% of the AC events. This study differs from many others in that it focuses specifically on the utility of CRP in AC screening.

Since AC is a lethal disease, we attempted to develop a more sensitive prediction model. The presence of AC was defined as a serum sodium level < 137 mEq/L or CRP level > 1.3 mg/dL. This criterion yielded better sensitivity values than either serum sodium or CRP levels alone, without a decrease in specificity. Although the model is very simple, it could help to distinguish between patients with AC and those with chronic AI, with a high diagnostic accuracy. To the best of our knowledge, this is the first study to develop a prediction model for AC diagnosis**.** Conventional practices, such as the provision of intensive patient education programs and diagnostic screening using changes in biochemical parameters excluding CRP, are considered important in the facilitation of AC recognition. However, they cannot aid in the drastic improvement of AC-related prognoses^3,5^. To overcome these difficulties, there is an urgent need for a highly sensitive method to promptly remind general practitioners of AC presence.

Based on this prediction model, two events in individuals with secondary AI were not detected (i.e., false-negative). However, as hypoglycaemia was observed in one case and anaemia in the other, the physician is likely to have detected AC. In contrast, the model yielded five false-positive results (two events in individuals with primary AI and three events in those with secondary AI). Although all these individuals showed substantial improvements in serum sodium and CRP levels in the chronic phases, four of the five individuals continued to show mild-to-moderate CRP level elevations of unknown aetiology, and the serum sodium level in the remaining patient was 136 mEq/L.

The main limitations of this study are the relatively small number of enrolled patients and events, and its retrospective design. However, we used strict enrolment criteria for the exclusion of all cases in which either the AI or AC diagnosis was doubtful. Another major limitation of this study was the use of data from the same individuals in a stable period during glucocorticoid replacement therapy as a control. This meant that the diagnostic accuracy of our prediction model was calculated in a study population with an AC incidence of 50%. Thus, as both AI and AC are rare conditions, the degree of applicability of our model to the general population may be limited. However, as well-known risk factors for AC include a history of AI^12^, adrenal or pituitary surgery^13^, dose reduction or discontinuation of exogenous steroid use^14,15^ and immune checkpoint inhibitors^16^ or steroid synthase inhibitor use^17, 18^, the model can prove useful in the early diagnosis of AC in individuals with these conditions. In fact, AC onset in patients with at least one of the aforementioned risk factors accounts for 73.3% (66/90) of the events. Finally, as the reference range differs somewhat according to the assay method used, the optimal serum sodium level and/or CRP level cut-off value for AC screening may vary slightly according to the medical facility.

In conclusion, this is the first study to develop a simple and practical prediction model for AC. We found that the use of a combination of serum sodium levels < 137 mEq/L and CRP levels > 1.3 mg/dL had a sensitivity of 97.8% and specificity of 94.4% in AC diagnosis. A larger prospective study is needed to validate the clinical utility and accuracy of different biochemical markers in AC diagnosis.

## Methods

### Participants

**Figure 2:**
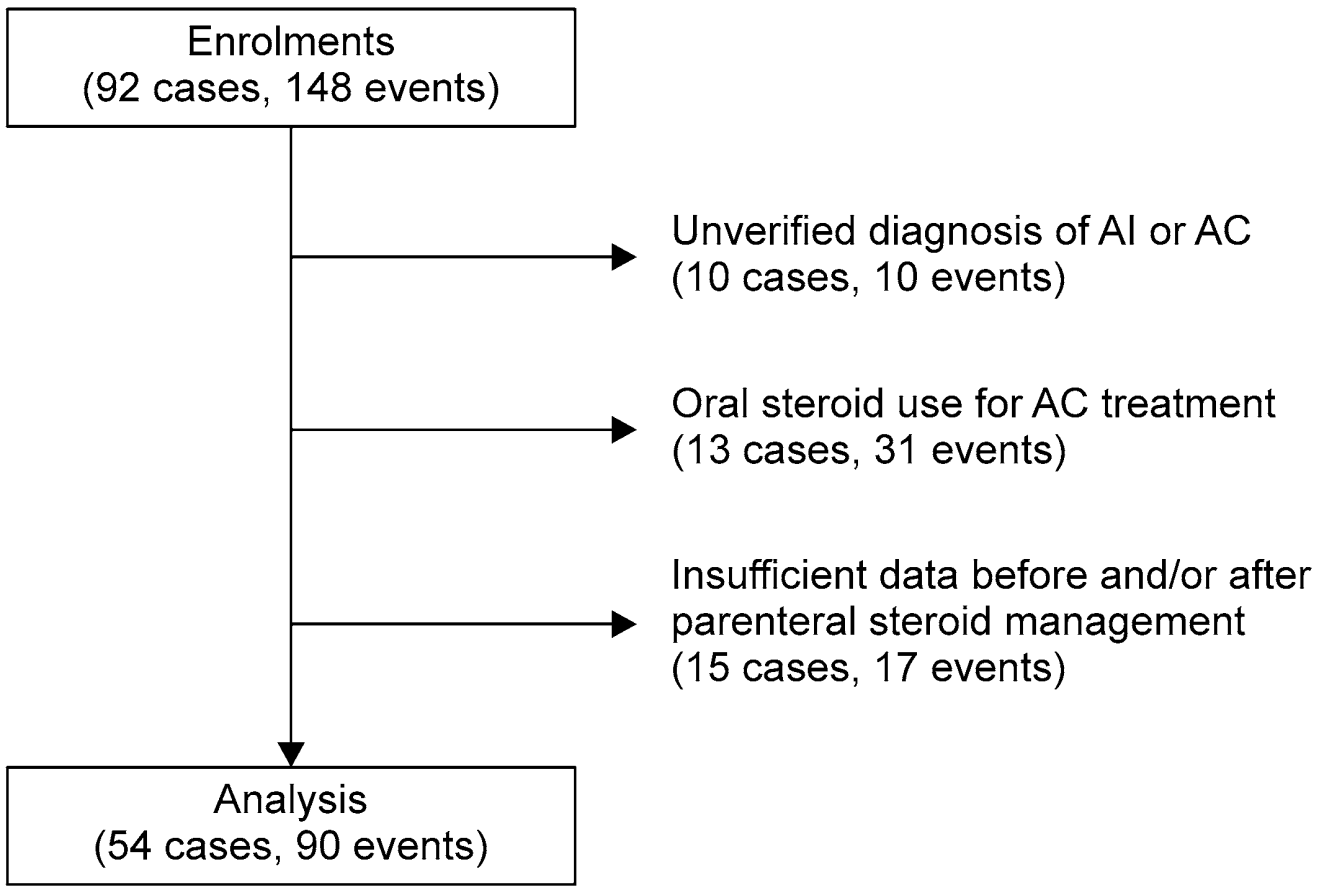
Flow diagram of patient enrolment. AC, adrenal crisis; AI, adrenal insufficiency.

We conducted a retrospective analysis of data from a multicentre collaborative study conducted at five referral centres in Japan. The study protocol was approved by the Human Ethical Committee of St. Marianna University School of Medicine (No. 4060) and the study was performed according to the clinical study guidelines published by the Ministry of Health, Labour and Welfare, Japan and the principles of the Declaration of Helsinki. Informed consent was waived by the Human Ethical Committee of St. Marianna University School of Medicine, because we collected and analyzed demographic and clinical data as these were used in an anonymized manner. First, we extracted data from the medical records of 92 inpatients (148 events) who were diagnosed with AI between November 2009 and June 2018 (Fig. 2). We then excluded patients with an unverified diagnosis of AI or AC (10 patients, 10 events), oral steroid use for AC treatment (13 patients, 31 events) or insufficient data before and/or after parenteral steroid management (15 patients, 17 events). The diagnosis of AC was confirmed if there was documentation of a worsening of the patient’s general condition with signs and symptoms of glucocorticoid and/or mineralocorticoid deficiency and at least one of the following conditions: hypotension (systolic BP < 100 mmHg); nausea or vomiting; severe fatigue; or documented hyponatraemia, hyperkalaemia, anaemia, or hypoglycaemia^19^. In addition, to further ensure accurate AC diagnosis, we only included patients whose symptoms were rapidly reversed by intravenous glucocorticoid administration^20^. After the exclusion of patients who did not meet the inclusion criteria, the final analytic dataset comprised data on 90 events in 54 patients.

The following data were collected at each event: patient demographics including age at admission, sex, AI aetiology and subtype (i.e., primary or secondary), precipitating factors for AC, previous diagnosis of AI and systolic BP. Data on patients’ biochemical profiles, including the serum sodium, potassium, glucose, haemoglobin, creatinine, eGFR and CRP values, were also collected. The eGFR was calculated using the following equation established for the Japanese population by the Japanese Society of Nephrology^21^.

$${\text{eGFR}}\,\left( {{\text{mL}}/{\min}/{1}.{\text{73 m}}^{{2}} } \right) = {194} \times {\text{serum creatinine}}^{{ - {1}.0{94}}} \times {\text{age}}^{{ - 0.{287}}} ( \times 0.{\text{739 for female patients}})$$

We chose these parameters for the development of a prediction model for AC as almost all physicians working in emergency units and general practitioners (i.e. non-endocrinologists) measure them in daily clinical practice and changes in biochemical parameters, as determined by laboratory tests, are often associated with AC^22^. Serum creatinine and eGFR were included in the analysis as renal function may have an effect on serum electrolyte levels. Data on all the predictors were collected both at AC onset (acute phase) and during the stable period during the administration of glucocorticoid replacement therapy (chronic phase). Patients were defined as being in the chronic phase if they received optimal glucocorticoid and/or mineralocorticoid replacement for at least three months within one year before and after the onset of AC. The presence of hyponatraemia (< 136 mEq/L in four centres, < 138 mEq/L in one centre) and CRP level elevations (> 0.3 mg/dL in four centres, > 0.14 mg/dL in one centre) were defined according to the reference range of each hospital. Sex-specific reference values were used for the determination of anaemia (Hb in men, < 13.7 g/dL in all five centres; and Hb in women, < 11.2 g/dL in four centres, and < 11.6 g/dL in one centre) and elevations in the serum creatine levels (men: > 1.04 mg/dL in four centres, > 1.07 mg/dL in one centre; women: > 0.74 mg/dL in four centres, > 0.79 mg/dL in one centre). Hyperkalaemia was defined as a level > 5.0 mEq/L, which has previously been reported to be useful in Addison’s disease diagnosis^23^. Hypoglycaemia was defined as a plasma glucose level < 70 mg/dL^24^. Low eGFR was defined as a value < 60 mL/min/1.73 m^2^^25^.

### Assay methods

Serum sodium and potassium levels were measured using ion-selective electrodes (Nihon Denshi, Co. Ltd., Tokyo, Japan; or Hitachi High-Technologies Corp., Tokyo, Japan). Plasma glucose levels were measured using glucokinase (LSI Medience Corp., Tokyo, Japan) or hexokinase (Hitachi High-Technologies Corp.) glucose-6-phosphate dehydrogenase methods. Hb levels were measured using Hb cyanide (Beckman, CA, USA) or SLS-Hb detection methods (Sysmex Corp., Tokyo, Japan). Serum creatinine levels were measured using Jaffe’s method (Beckman Coulter, CA, USA) or chromogenic enzyme assay (Hitachi High-Technologies Corp.). CRP levels were measured using latex photometric immunoassay (LSI Medience Corp.) or latex agglutination turbidimetry (Hitachi High-Technologies Corp.).

### Statistical analysis

Results are presented as mean ± standard error, frequencies, and proportions, unless otherwise stated. Clinical parameters during the acute (AC) and chronic (AI) phases were compared using paired Student’s *t*-test for continuous variables with a normal distribution or Wilcoxon signed-rank test for continuous variables with a non-normal distribution. Factors showing significant differences between the two phases were included in the multivariate logistic regression models. For all comparisons, AC, as defined above, was used as the comparator (true positive). Binary logistic regression analysis was performed for the identification of clinical parameters in the acute phase that were independently significantly associated with AC presence using the values from the chronic phase as comparators. The results were reported as ORs with their 95% CIs. ROC analysis was used to determine the test characteristics of the different variables predictive of diagnosis and provide the best sensitivity and specificity value combination. Statistical analyses were performed using EZR (Saitama Medical Center, Jichi University, Saitama, Japan). The level of significance was set as *P* < 0.05.

## Data Availability

Restrictions apply to the availability of data generated or analysed during this study for the preservation of patient confidentiality or because they were used under license. The corresponding author will, on request, detail the restrictions and any conditions under which access to some data may be provided.
